# Editorial: Cancer risk in patients with acromegaly – is extensive screening needed?

**DOI:** 10.3389/fendo.2024.1503633

**Published:** 2024-10-28

**Authors:** Raluca Alexandra Trifănescu, Jakob Dal

**Affiliations:** ^1^ Department of Endocrinology, “Carol Davila” University of Medicine and Pharmacy, Bucharest, Romania; ^2^ First Endocrinology Department, “C. I. Parhon” National Institute of Endocrinology, Bucharest, Romania; ^3^ Department of Endocrinology, Aalborg University Hospital, Aalborg, Denmark; ^4^ Steno Diabetes Center North Jutland, Aalborg, Denmark

**Keywords:** acromegaly, cancer, incidence, prevalence, screening, thyroid, colorectal

Acromegaly is a rare endocrine disorder characterized by excessive growth hormone (GH) and insulin-like growth factor I (IGF-I) production. Its symptoms include enlarged extremities and organs; complications are cardiovascular, respiratory, metabolic, musculoskeletal, neurological, benign and malign tumors (1–3). However, the extent to which acromegaly increases cancer risk and mortality remains controversial, as does the need for extensive screening. The most recent consensus statement did not establish whether colonoscopy should be performed in all patients at diagnosis, regardless of age (4). The previous guideline of Endocrine Society recommended a baseline screening colonoscopy, with follow-up every five years if polyps are present or IGF-1 levels are elevated (5). For other cancers associated with acromegaly, including thyroid cancer, screening should be performed according to guidelines for the general population (4, 6).

While GH may play a role in malignancy progression (enhancing promitogenic and anti-apoptotic properties), it does not seem to induce malignancy (7). Complications of acromegaly, such as increased intestinal size or diabetes are other risk factors for malignancy (8). However, the risk of malignancies in acromegaly remains controversial. Epidemiological studies show diverging results. Up to 2016, a modestly increased cancer risk was reported, especially for colon and thyroid neoplasms (9–12). Several studies have since then reported an increased cancer risk (10–16), while others have not (17) – see **Table 1**. A recent meta-analysis revealed an increased risk of thyroid, colorectal, brain, gastric, urinary, hematological, pancreatic, small intestine, and connective tissue cancer (18). From an epidemiological perspective, conflicting data could be explained by study limitations such as the low number of patients, surveillance bias, the lack of adjustment of confounding variables, the heterogeneity of control groups (general population/other pituitary adenomas), and inaccuracies in comparison older and more recent studies due to time-depended changes in life expectancy, modifications in cancer.

**Table 1:** Incidence of cancer in acromegaly patients in selected studies published in the last 10 years.

Author, year of publication	No of acromegaly patients	SIR for malignant tumors	Type of cancer	Notes
Xiao Z, 2023 (18)	Meta analysis of 19 studies 11494 patients	1.45 [1.2-1.75]	Thyroid SIR= 6.96 [2.51-19.33] Colorectal and anal SIR=1.95 [1.32-2.87] Brain and central nervous system SIR= 6.14 [2.73-13.84] Gastric SIR= 3.09 [1.47-6.50] Urinary SIR= 2.66 [1.88-3.76] Hematological SIR= 1.89 [1.17-3.06] Pancreatic and small intestine SIR= 2.59 [1.58-4.24] Connective tissue SIR= 3.15 [1.18-8.36]	No increase in hepatobiliary, respiratory, reproductive, skin, breast, or prostate cancer
Xiao ZH, 2023 (25)	117 (2011–2022)	3.29 [1.42-6.94]	Colorectal SIR=16.67 [4.45-42.67] Thyroid SIR= 14.29 [1.73-51.60]	
Xiao T, 2023 (13)	1738 (2012–2020)	2.81 [2.18-3.57]	Thyroid SIR=21.42 [13.74-30.08] Colorectal SIR=3.17 [1.37-6.25]	
Durmus ET, 2022 (14)	214	4.78 in women [2.43-8.53] 8.97 in men [5.51-14.7]	Thyroid, colorectal, breast, kidney, gastric, and testicular	
Esposito D, 2021 (15)	1296 (1987–2017)	1.3 [1.1-1.5]	Colorectal and anal SIR= 1.5 [1.0-2.2] Renal and ureteral SIR= 4 [2.3-6.5]	SIR for respiratory system, brain, breast, prostate cancer not increased
Ucan B, 2021 (24)	280	0.8 in men [0.5-1.1] 1.0 in women [0.8-1.3]	Cancer prevalence 6.8% Thyroid 3.2%	
Dal J, 2018 (10)	529 (1978–2010)	1.1 [0.9-1.4]	Colorectal SIR= 1.4 [0.7-2.6] Prostate SIR= 1.4 [0.6-2.6] Hematological SIR= 1.3 [0.4-3] Breast SIR= 1.1 [0.5-2.1] Urinary tract SIR = 1.0 [0.3-2.4]	
Terzolo M, 2017 (12)	1512 (1980–2002)	1.41 [1.18-1.68]	Colorectal SIR= 1.67 [1.07-2.58] Kidney SIR= 2.87 [1.55-5.34] Thyroid SIR= 3.99 [2.32-6.87]	
Maione L, 2017 (16)	999 (1977–2012)	1.34 in men [0.94-1.87] 1.24 in women [0.77-1.73]		SMR= 1.05 [0.70-1.42]. Most deaths were due to cancer.
Petroff, 2015 (17)	446	0.75 [0.55-1.0]		Not significantly higher for colorectal, breast, thyroid, prostate, and lung cancers.

The current Frontiers in Endocrinology Research Topic presents real-life data from several large cohorts of acromegaly patients. Advances in acromegaly treatment have led to a greater potential to achieve disease control by decreasing the GH and IGF-I burden and improving follow-up care which may reduce cancer risk (19). Furthermore, the overall increased awareness of acromegaly, with decreasing diagnostic delay, and increasing incidence rate of patients with a milder phenotype of acromegaly, possibly also affect the cancer risk (20–22).

Data from the Danish nationwide AcroDEN cohort (739 patients treated since 1990) demonstrate improved diagnostics, with a continual increase in the likelihood of being diagnosed and treated for conditions such as diabetes, heart disease, sleep apnea, joint disease, and osteoporosis (Rosendal et al.). Also, an increased proportion of patients achieving hormonal disease control was reported (69% to 88%). The risk of being diagnosed with cancer did in contrast not significantly change, with an overall cancer risk of 1.1 (10) and a mortality rate of 1.3, mainly attributed to cardiovascular mortality (9, 10).

Rolla et al. assessed acromegaly’s complications at a referral center in Poland. In a series of 179 patients (1976–2018), 40% were cured by surgery, and 31% were pharmacologically controlled. During 496 hospitalizations, 43 colonoscopies were performed, and 21 colonic polyps were discovered (11.7% of cases). Despite the frequent use of thyroid ultrasound (198/496 hospitalizations) and a high proportion of goiter (52%), only two cases of thyroid cancer were diagnosed (24). A similar low risk of thyroid cancer in acromegaly was reported from the AcroDEN cohort (10).

The effect of modern treatment was further reported by *Galoiu S* et al. from a referral center in Romania, including 399 acromegaly (2001–2022). The surgical cure rate was 31% and the pharmacologically controlled was 22%. The standardized mortality rate (SMR) was 1.18, and decreased (from SMR=1.25) to a level comparable to the reference population (SMR=1.09) in patients diagnosed after 2008. Males had a lower mortality ratio (SMR=0.99), compared to females with acromegaly (SMR=1.63) (Găloiu et al.). Several studies have reported a similar trend, with decreasing mortality and a shift in the causes of death resembling those of the reference population (21). In the AcroDEN cohort, the mortality was slightly increased (SIR=1.3), whereas cancer-specific mortality was not (10).

The incidence of benign tumors is increased in acromegaly (SIR=2.4) (15). Guo et al. conducted a systematic review of 24 studies on the prevalence of meningiomas in patients with acromegaly or those exposed to exogenous GH therapy. Meningiomas occurred either synchronous or metachronous with acromegaly; no significant correlation was found between GH/IGF-1 levels and meningioma size; some patients with acromegaly and meningiomas were treated with radiotherapy, which is known to increase the risk of developing a second brain tumor (23) In four cases of acromegaly with meningiomas, without previous radiotherapy, there was a family history of pituitary adenomas and cancers. The authors suggest that hereditary cancer syndromes might play a role in the co-occurrence of acromegaly and meningiomas (Guo et al.). Whereas skin cancer risk is not increased in acromegaly (18), skin lesions were more frequently observed in acromegaly compared to patients with non-functioning pituitary adenomas. There was an improvement of these skin lesions three months after surgery in younger patients and patients who presented with the highest GH levels (Guo et al.).

In summary, acromegaly is associated with an increased risk of certain types of cancers, although the specific evidence remains controversial. Early diagnosis and advances in the treatment of acromegaly improved disease control and decreased mortality rates. Current data do not justify extensive cancer screening beyond standard guidelines, although clinical attention is crucial, especially in elderly patients (13, 24), in patients with a longer disease duration (14, 25), and in acromegaly patients with diabetes mellitus (25) Large studies with long follow-up are needed to further understand the impact of GH excess on cancer risk and to refine screening protocols.
